# Metabolic Dynamics During Loquat Fruit Ripening and Postharvest Technologies

**DOI:** 10.3389/fpls.2019.00619

**Published:** 2019-05-24

**Authors:** Jianghua Cai, Tong Chen, Zhanquan Zhang, Boqiang Li, Guozheng Qin, Shiping Tian

**Affiliations:** ^1^ Key Laboratory of Plant Resources, Institute of Botany, Chinese Academy of Sciences, Beijing, China; ^2^ College of Life Science, University of Chinese Academy of Sciences, Beijing, China; ^3^ Key Laboratory of Post-Harvest Handing of Fruits, Ministry of Agriculture, Beijing, China

**Keywords:** loquat fruit, metabolites, development, ripening, postharvest treatment

## Abstract

Loquat is an important fruit widely cultivated worldwide with high commercial value. During loquat fruit development, ripening, and storage, many important metabolites undergo dramatic changes, resulting in accumulation of a diverse mixture of nutrients. Given the value of loquat fruit, significant progresses have been achieved in understanding the metabolic changes during fruit ripening and storage, as well as postharvest technologies applied in loquat fruit in recent years. The objective of the present review is to summarize currently available knowledge and provide new references for improving loquat fruit quality.

## Introduction

Loquat (*Eriobotrya japonica* Lindl.) is a subtropical evergreen fruit tree originated in south China. It has been cultivated for more than 2000 years in China and is now widely cultivated in over 30 countries around the world (Lin et al., 1999), and China is the largest producer with a growing area and production. Loquat blooms in autumn and early winter, and its fruit ripens in early summer when other fruits are in off-seasons. Loquat fruit is favored by consumers for its attractive appearance, juicy taste, and rich nutrients. Moreover, it is an important source of soluble fiber, vitamins, carotenoids, antioxidants, and minerals including calcium, potassium, phosphorus, and magnesium that are essential to human body (Tian et al., 2007). In addition, loquat has numerous medical functions previously recorded in “Compendium of Materia Medica,” which plays important roles in regulating blood pressure; stimulating circulatory system; lowering risk of cancer; treating inflammation; preventing diabetes; soothing the respiratory system; improving immune system, digestion, skin health, and eye vision; and fighting against viruses (Kumar et al., 2014). Therefore, loquat is also considered as a health fruit.

**Figure 1:**
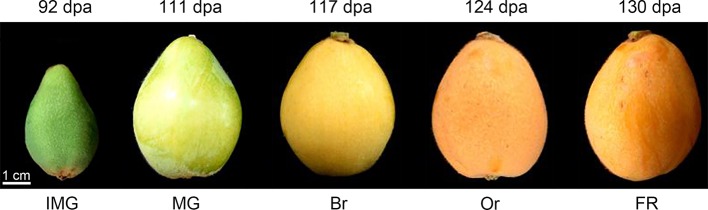
Phenotypes of loquat fruit during different developmental stages. The whole development process can typically be divided into five stages, including immature green (IMG) at 92 days postanthesis (dpa), mature green (MG) at 111 dpa, breaker (Br) at 117 dpa, orange (Or) at 124 dpa, and fully ripe (FR) at 130 dpa. Bar = 1 cm.

Loquat fruit is classified as non-climacteric fruit, since there is no sudden rise in respiration rate and ethylene production during fruit ripening (Alos et al., 2017). Loquat fruit requires 4–5 months from blossom to fully ripe, which could be divided into five stages: immature green (IMG), mature green (MG), breaker (Br), orange (Or), and fully ripe (FR) (Figure 1), with reference to fruit development and ripening process in the model species, *Solanum lycopersicum*. Loquat fruit ripening is a complex and precisely regulated process involving numerous physiological and chemical changes in primary and specialized metabolites, including pigments, sugars, organic acids, and phenolic compounds (Tian et al., 2007). Therefore, the understanding toward metabolic changes in loquat fruit may be helpful for improving fruit quality and values. During the past several decades, many advances have been achieved in revealing the variations in metabolites during fruit development, ripening, and postharvest storage. This review will focus on variations in major metabolites during loquat fruit development ripening and postharvest storage and also summarize postharvest technologies currently available for loquat industry.

## Changes in Metabolites of Loquat During Fruit Development and Ripening

### Carotenoid Metabolism

**Figure 2:**
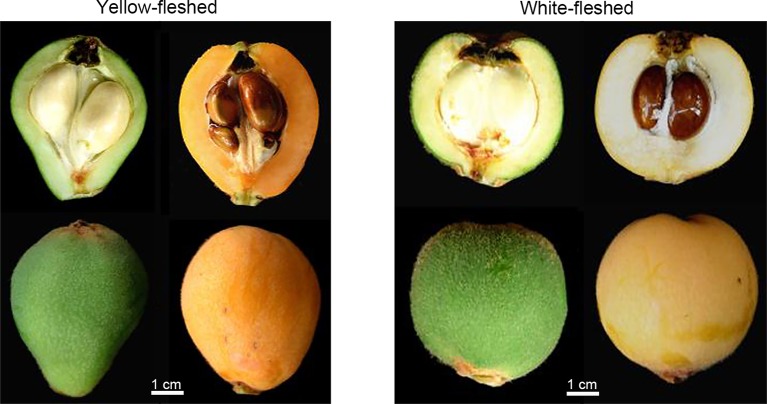
Phenotypes of yellow-fleshed and white-fleshed loquat fruit at fully ripening. Bar = 1 cm.

**Figure 3:**
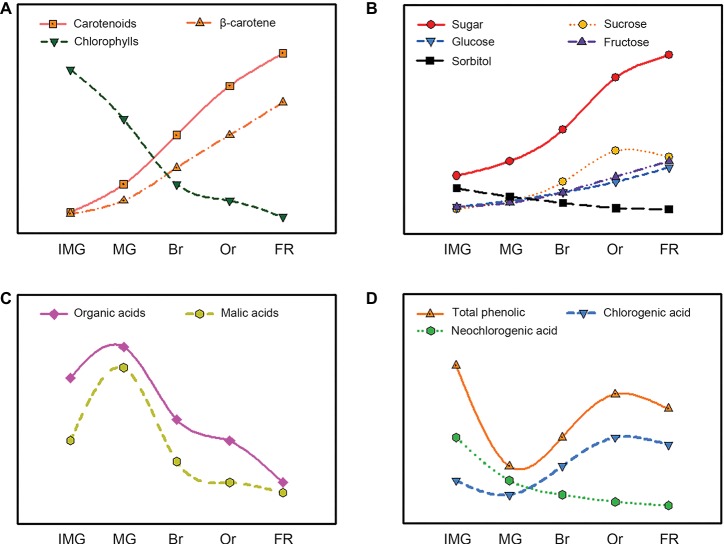
Evolution of the carotenoids **(A)**, sugars **(B)**, organic acids **(C)**, and phenolics **(D)** content during loquat fruit development and ripening.

Carotenoids are important pigments associated with yellow-to-red color ranges. They play vital roles in determining fruit color, which is a critical factor for loquat fruit quality (Gross et al., 1973). According to the color of fruit fresh, loquat fruit can be divided into two groups: yellow-fleshed and white-fleshed (Figure 2). The composition and content of carotenoids accumulated in yellow- and white-fleshed loquat fruit are significantly different. Yellow-fleshed fruit showing orange or red color contain high levels of carotenoids, whereas white-fleshed fruit exhibiting white color have a low level. Zhou et al. (2007) found that 12 yellow-fleshed fruits had a higher content 214.50–475.22 μg/g dried weight (DW) than 11 white-flesh cultivars 91.52–202.28 μg/g DW. Reported that nine yellow-fleshed fruits had much higher level β-carotene 11.55–15.69 μg/g fresh weight (FW) than two white-fleshed fruits. Conversely, the percentage of lutein in yellow-fleshed fruits was much lower than that of white-fleshed fruit. Moreover, there are significant differences in content and composition of carotenoid between flesh and peel in loquat fruit. Beta-carotene and lutein are the most abundant carotenoids in the peel and accounted for about 60% of the total colored carotenoids in 23 yellow- and white-fleshed cultivars, whereas more than a half of the colored carotenoids in the flesh were beta-carotene (Zhou et al., 2007). Thirty-two types of carotenoids were reported in peel and only 18 were identified in flesh (Hadjipieri et al., 2017). In general, the most obvious changes in size and color occur during loquat fruit development and ripening. Immature and mature green loquat fruit contain a higher level of chlorophylls with a few carotenoids, which was similar to those in leaves. During fruit ripening, chlorophylls are rapidly degraded, whereas massive carotenoids are synthesized, resulting in changes in fruit color from green to yellow (Figure 3A). Total amount of carotenoids in yellow-fleshed cultivar and white-fleshed cultivar did not have difference at mature green stage, but increased dramatically during ripening, leading to a significant difference in the two cultivars (Zhang et al., 2016a). Levels of beta-carotene in peel and flesh of “Zaozhong 6” increased gradually and reached the peak levels at fully ripen stage (Zhang et al., 2016a). These data indicate significant changes in carotenoid content and compositions during loquat fruit development and ripening.

Plenty of researches have proven that the transcriptional regulations on the carotenoid biosynthesis pathway play crucial roles in the differential accumulation of carotenoids in plants. Over the past several decades, numerous attempts have been made to elucidate the mechanism for the differences between yellow-fleshed and white-fleshed loquat fruit, especially in the expression of carotenoid biosynthesis genes. Sun et al. (2013) reported that the transcriptional downregulation of β-carotene 3-hydroxylase gene (*HYB*) was the main reason for lacking of β-carotene in the white-fleshed mutant. Fu et al. (2011) proved that the lower expression of phytoene synthase 1 (*PSY1*), chromoplast-specific lycopene β-cyclase (*CYCB*), and β-carotene hydroxylase (*BCH*) was closely correlated with the lower level of carotenoid in white-fleshed “Baisha” fruit. Similarly, the expression of *PSY1*, ζ-carotene desaturase (*ZDS*), *CYCB*, and *BCH* in the peel and flesh of “Obusa” were upregulated during fruit ripening (Hadjipieri et al., 2017). These results suggest that the expression of *EjPSY*, *EjCYCB*, and *EjBCH* may play important roles in regulating carotenoids synthesis during loquat fruit ripening. PSY is a key enzyme well received as a “star player” in carotenoid biosynthesis. Numerous studies have been conducted to regulate carotenoid content *via PSY* (Fraser et al., 2007; Fantini et al., 2013). In loquat, four *PSY* genes, *EjPSY1*, *EjPSY2A*, *EjPSY2B*, and *EjPSY3*, have been characterized, and they exhibited different expression patterns. *EjPSY1* mainly expressed in leaves, and the peel of loquat fruit, *EjPSY2A*, showed high expression in the peel and flesh during fruit ripening, whereas *EjPSY2B* only expressed in leaves and *EjPSY3* displayed a low expression in all the tissues examined (Fu et al., 2014). These results suggested that *EjPSY1* and *EjPSY2A* were responsible for carotenoid accumulation in peel and flesh, respectively. A mutant *EjPSY2Ad*, which loses the C-terminal catalytic domain and has no PSY catalytic activity, was found in all seven white-fleshed cultivars, explaining why white-fleshed fruit have lower level of carotenoids. *EjPSY3* contains alternatively spliced forms in “Luoyangqing” and “Baisha” and has no function in carotenoid accumulation (Fu et al., 2014). In addition, virus-induced gene silencing-mediated suppression of *EjPSY* resulted in decreased carotenoid content in loquat fruit (Hong et al., 2018), indicating that *PSY* gene positively regulated carotenoid accumulation in loquat fruit.

Carotenoids are synthesized and accumulated in plastid during fruit ripening, and it has been demonstrated that plastids play important roles in regulating carotenoid accumulation (Lu et al., 2006) and also undergo significant changes during the differentiation of chloroplasts into chromoplasts during loquat fruit ripening. Some studies showed that numerous chromoplasts existed in the peel and flesh of yellow-fleshed “Luoyangqing” fruit at fully ripe stage, while less chromoplasts in smaller size were detected in the peel of white-fleshed “Baisha” fruit and no chromoplast was found in the flesh of “Baisha” fruit (Fu et al., 2011). Further investigation revealed that carotenoids were mainly deposited in the lipid globules in chromoplasts of “Luoyangqing” and “Baisha” peel and presented in crystalline form in the flesh of “Luoyangqing” fruit (Fu et al., 2011). Therefore, abnormal chromoplast development may be responsible for the lack of carotenoid accumulation in white-fleshed loquat. In addition to these, genes involved in chloroplast differentiation or carotenoid sequestration also participated in carotenoid accumulation. ORANGE (*OR*) encodes a DnaJ protein and is considered to be a master regulator for normal differentiation of chromoplast in cauliflower and melon fruit (Lu et al., 2006; Tzuri et al., 2015). The *OR* homologous gene was isolated from loquat, and the transcript level of *EjOR* was slightly higher in “Luoyangqing” than that in “Baisha” during fruit ripening (Fu et al., 2011), indicating that *OR* gene might function in regulating carotenoid accumulation in loquat fruit.

### Sugar Metabolism

Sugar and organic acid are major soluble components in ripe fruits and have vital roles in fruit taste and flavor, which are key indicators of fruit quality (Zhu et al., 2013; Liu et al., 2016). Sugar and acid content as well as the ratio of them are the important index of flavor of fruits. Numerous studies showed that fructose, sucrose, glucose, and sorbitol were major sugars in ripe loquat fruit, but the ratio of these ingredients varied among different cultivars. Hirai (1980) showed that sucrose was the most abundant sugar in the ripe “Tanaka” loquat, whereas sorbitol predominated in the young fruit. Ding et al. (1998a) reported that the major sugars in “Mogi” loquat fruit were fructose, sucrose, glucose, and sorbitol. Glucose and fructose serve as the dominant sugars and constitute at least 80% of the total sugar in 12 cultivars of loquat fruit, whereas sucrose and sorbitol were less abundant (Xu and Chen, 2011). Similarly, Toker et al. (2013) reported that glucose and fructose acted as the primary sugar compositions and accounted for more than 91.42% in 15 cultivars of loquat fruit in Turkey. These studies suggest that significant differences in the composition of sugars were observed among various cultivars, which may be attributed to different genetic backgrounds and cultivation environment. During loquat fruit development and ripening, sugar content is relatively slow at early stages but increases rapidly during fruit expanding period and further slows down at breaker stages (Figure 3B; Hirai, 1980; Serrano et al., 2003; Ni et al., 2011). Sorbitol, a primary sugar in young loquat fruit, decreases with fruit development, whereas sucrose, glucose, and fructose increase sharply with fruit ripening, and sucrose accumulates faster than glucose and fructose and then becomes the major sugar in ripe fruit (Serrano et al., 2003; Wu et al., 2015). Sucrose contents in yellow-fleshed “Dahongpao” and white-fleshed “Ninghaibai” are unchanged during fruit development, whereas sorbitol contents decreased rapidly with fruit development (Chen et al., 2010), indicating that fructose and glucose contents increase dramatically during ripening and become the dominant sugars in loquat fruits.

According to the types of production, sugar metabolism could be sorted into sucrose, sorbitol, and hexose metabolism in fruits (Kanayama, 2017). Sucrose phosphate synthase (SPS), sucrose synthase (SS), and invertase (IN) are key enzymes involved in sucrose metabolism (Bruneau et al., 1991; Sturm, 1999). Sorbitol-6-phosphate dehydrogenase (S6PDH), sorbitol dehydrogenase (SDH), and sorbitol oxidase (SOX) are key enzymes responsible for sorbitol metabolism (Sakanishi et al., 1998; Iida et al., 2004). All these enzymes play important roles in the sugar metabolism during fruit ripening. Abnasan-Bantog et al. (1999) found that the activities of SDH, SPS, SS, and AIV enzymes in “Mogi” fruit were low at early stages of fruit development but increased dramatically during fruit ripening stages simultaneously with sugar accumulation. However, the activity, protein abundance, and transcript level of S6PDH increased during loquat development and decreased during ripening in parallel with NAD^+^-SDH (Abnasan-Bantog et al., 2000), indicating that sorbitol may be synthesized in loquat fruit. Ni et al. (2009) reported that the changes in the activities of SS and SPS were positively correlated with the dynamics in sucrose accumulation, and thus, they proposed that SS and SPP were key enzymes in the regulation of sucrose accumulation in “Qingzhong,” “Bahong,” and “Jidanbai” fruit. Chen et al. (2010) demonstrated that the activities of IN, SS, and SDH decreased at early stages of fruit development but increased at later stages of fruit ripening in white-fleshed cultivar “Ninghaibai” and yellow-fleshed cultivar “Dahongpao” and that the activities of IN and SS cleavage in yellow-fleshed fruit were lower than those in white-fleshed fruit at late stages of fruit development. Currently, most attempts focus on patterns of sugar accumulation and activities of related enzymes, and much less are known about the transcriptional changes of sugar metabolism genes. Up to now, the genes encoding SS, SPS, SDH, VIN, sorbitol transporter protein, and fructokinase have been cloned. Wang et al. (2015a) found that the transcript level of *EjVIN* was highest in the early stages of fruit development and ripe fruit and could be induced by exogenous fructose or glucose treatment. Further analyses showed that overexpression of loquat *EjVIN* in tobacco decreased the sucrose level, indicating that *EjVIN* played important role during early development stage of loquat fruit. Song et al. (2016) showed that the transcript levels of *EjSPS1, EjSPS2*, and *EjSS-C* in “Jiefangzhong” fruit increased gradually during fruit development and reached the peaks at fully ripe stage, whereas the transcript level of *EjSS-S* was high at early stage and dramatically decreased with fruit development. These results indicated that *EjSPS1, EjSPS2*, and *EjSS-C* had vital roles in promoting sugar accumulation in “Jiefangzhong” loquat fruit. Li et al. (2016a,b) cloned *NAD^+^-SDH* gene from the yellow-fleshed cultivar “Dongting” loquat, which is a white-fleshed bud mutant, and proved that three SNP loci were presented in the *NAD^+^-SDH* gene from the mutant fruit, leading to excessive conversion of sorbitol into fructose at maturation stage. These data suggest that the sugar content in loquat fruit may also be influenced by cultivation measures and exogenous substances during fruit development and ripening.

### Organic Acid Metabolism

Organic acids are also determiners in fruit flavor and can be used as substrates for respiration. Numerous researches showed that malic, tartaric, quinic, citric, succinic, fumaric, and oxalic acids were predominant organic acids in ripe loquat fruit, but their composition and contents in loquat fruit varied among cultivars (Toker et al., 2013). They found that malic acid was the primary acid, followed by tartaric, succinic, oxalic, and citric acids in ripe fruits of 12 loquat cultivars in Turkey. Organic acid in loquat fruit increased at the early stages of fruit growth, decreased with fruit ripening, and reached the lowest at full ripen (Figure 3C; Serrano et al., 2003; Chen et al., 2009). Likewise, malic acid was the predominant organic acid in unripe loquat fruit and exhibited a similar change as the total organic acid during loquat fruit development and ripening, whereas citric acid, succinic acid, and ascorbic acid contents were much lower than malic acid, which decreased to the lowest at the full ripening stages (Serrano et al., 2003). Chen et al. (2009) also reported that the malic acid and citric acid contents increased during the early stages of fruit development and decreased at the later stages in “Changhong 3” and “Jiefangzhong,” but quinic acid was the abundant in loquat fruit flesh at the early stages of fruit development and decreased with the fruit development and ripening.

Organic acid metabolism is a complex process involving biosynthesis, transport, storage, and degradation of multiple organic acids, which keeps the contents of organic acids in balance. Given the importance and content of malic acid, significant progresses have been achieved in revealing malic acid metabolism in fruits. Malic acid content is positively correlated with NAD^+^-dependent malate dehydrogenase (NAD-MDH) activity and negatively with NADP^+^-dependent malic enzyme (NADP-ME) activity (Chen et al., 2009), suggesting that NAD-MDH and NADP-ME might play important roles in malate biosynthesis and degradation. In addition, genes encoding *EjPEPC*, *EjNADP-ME*, *EjcyNAD-MDH*, *EjmNAD-MDH*, *EjV-ATPase A*, and *EjV-PPiase* were cloned by Yang et al. (2011), and they found that the transcript level of *EjNADP-ME* in the high-acid cultivar was significantly higher than that in the low-acid cultivar, whereas the expression of *EjmNAD-MDH*, *EjV-ATPase A*, and *EjV-PPiase* displayed opposite patterns. The expression of *EjNADP-ME*, *EjmNAD-MDH*, *EjV-ATPase A*, and *EjV-PPiase* exhibited similar patterns in both cultivars, whereas the expression patterns of *EjPEPC* and *EjcyNAD-MAD* were different (Yang et al., 2011), indicating that these enzymes may play a vital role in regulating malic acid accumulation in loquat fruit.

### Phenolic Metabolism

Phenolic compounds serve as important secondary metabolites, which are crucial for defense response, anti-oxidation, color formation, seed dormancy, programmed cell death, and responses to abiotic stresses (Vogt, 2010). Up to now, hydroxybenzoic acid, hydroxycinnamic acid, flavonol, flavanone, flavone, lignin, and other phenolic compounds have been identified in loquat fruit. However, the total amount and the compositions of phenolic compounds exhibited great differences in different tissues of loquat fruit (Koba et al., 2007). The highest total phenolic content was found in seeds, followed by peel and flesh (Pande and Akoh, 2010). The composition and content of phenolic compounds in ripe loquat fruit also vary greatly among different cultivars. Ding et al. (2001) analyzed the composition of phenolic compounds in seven Japanese loquat cultivars and found the contents of phenolic compounds varied from 81.8 to 173.8 mg/100 g FW. Ercisli et al. (2012) showed that the total phenolic contents of seven Turkish cultivars of loquat fruit ranged from 140 to 253 μg g^−1^ FW. Xu et al. (2014a) reported that chlorogenic acid, neochlorogenic acid, 4-*O*-caffeoylquinic acid, protocatechuic acid, and 4-hydroxybenzoic acid were the primary phenolic compounds in ripe loquat fruit. Chlorogenic acid was considered to be the major phenolic compound and accounted for at least 75% of total phenolic in the dried fruit of seven loquat cultivars in China (Zhang et al., 2015).

During loquat fruit development and ripening, the total phenolic contents first decreased sharply and then increased dramatically (Figure 3D; Ding et al., 2001). Neochlorogenic acid was the major phenolic in young fruit and its content decreased with fruit ripening, whereas chlorogenic acid content underwent similar changes and became the predominant phenolic in ripe fruit of “Mogi” and “Tanaka” (Ding et al., 1998b, 2001). The increases in total phenolic and chlorogenic acid can be recognized as indicators for loquat fruit ripening. Moreover, the composition of phenolics in young loquat fruit is more diversified than those in ripe fruit. More than 10 types of phenolics were detected in young “Mogi” fruit, whereas only 7 types were in ripe loquat (Ding et al., 2001). Given the scientific importance of chlorogenic acid, the activities of enzyme involved in chlorogenic acid biosynthesis were extensively investigated (Clifford et al., 2017). Chlorogenic acid is a product of phenylalanine metabolism and its biosynthesis pathways are well illustrated in plants (Niggeweg et al., 2004). Phenylalanine ammonia lyase (PAL), 4-coumarate: CoA ligase (CL), and hydroxycinnamoyl CoA: quinate hydroxycinnamoyl transferase (HQT) are key enzymes involved in chlorogenic acid biosynthesis (Gramazio et al., 2014). The activities of PAL and HQT in loquat fruit was high initially, but decreased to the lowest levels, and then increased to the peaks at 1 week prior to harvest (Ding et al., 2001). The changes of these enzyme activities were well correlated with the variations in chlorogenic acid during development and ripening of loquat fruit.

## Changes in Metabolites of Loquat Fruit During Storage

Loquat fruit is consumed largely as fresh fruit, which should be harvested at the eating-ripe stage and transported to consumers. After harvest, respiration is the most important physiological process in loquat fruit and causes the fruit nutrients to be gradually degraded, eventually leading to deterioration in fruit quality and decay (Tian et al., 2007; Pareek et al., 2014). Loquat belongs to a non-climacteric fruit and has ethylene production at a low level during postharvest storage (Blumenfeld, 1980). The respiration and ethylene production gradually declined after harvest, which can be dramatically suppressed at low temperature (Ding et al., 1998a; Alos et al., 2017). Ethylene has been shown to play vital roles in regulating climacteric fruit ripening *via* ethylene biosynthesis and signaling (Liu et al., 2015a). Although numerous studies showed that ethylene was not necessary for the ripening of non-climatic fruit, exogenous ethylene treatment could also influence ethylene emission of loquat fruit. Treatment with exogenous ethylene increased ethylene release of “Luoyangqing” fruit and enhanced fruit browning, whereas 1-methlcyclopropene (1-MCP) treatment inhibited browning by lowering lipoxygenase (LOX) and polyphenol oxidase (PPO) activities and reducing O^2−^ accumulation and oxidation of polyphenols (Cai et al., 2006a). Exogenous ethylene could also influence the ripening of loquat *via* ethylene signaling, which depends on loquat cultivars. Wang et al. (2009) showed that the transcript abundance of the genes involved in ethylene signaling decreased during fruit ripening, but ethylene treatment could strongly induce ethylene response sensor 1a (*EjERS1a*), ethylene response sensor 1a (*EjERS1b*), constitutive triple response 1 (*EjCTR1*), and ethylene response factor 3 (*EjERF3*) expression in “Luoyangqing.” Alos et al. (2017) found that the transcript levels of 1-aminoacyclopropane 1-carboxylate synthase 1 (*EjACS1*), 1-aminoacyclopropane 1-carboxylate oxidase 1 (*EjACO1*), *EjCTR1*, and ethylene-insensitive 3-like 1 (*EjEIL1*) were significantly increased during storage periods, and exogenous ethylene treatment did not affect the expression of these genes involved in ethylene biosynthesis and signaling, whereas 1-MCP significantly inhibited the expression of *EjACS1*, *EjACO1*, *EjACO2*, *EjERS1a*, and *EjCTR1.*

Loquat fruit has a short postharvest life (no more than 10 days) at normal temperature and undergoes a series of physiological disorders, including internal browning, adherence of the peel and flesh, dry pulp tissue, and decay (Ding et al., 2002; Tian et al., 2007). In addition, many metabolites also show significant changes during storage. Total sugar decreased sharply, and the rate of declination was significantly negatively correlated with the storage temperature during storage. Sucrose, fructose, glucose, and sorbitol are the main sugars in the ripe loquat fruit. Among these sugars, sucrose declined rapidly, while fructose, glucose, and sorbitol showed slight changes during storage at 20°C (Ding et al., 1998a). Low temperature storage is effective to maintain loquat fruit quality. Wei et al. (2017) reported that “Jiefangzhong” loquat fruit stored at 0°C exhibited higher glucose and fructose content than those stored at 5°C, which should be attributed to the increases in the activities of AI, NI, SPS, and SS. Total acid content exhibited a decrease during storage. The primary organic acids in ripe loquat fruit is malic acid, which is represented about 90% of total acids. During storage, the concentration of malic acid declined dramatically and displayed a negative correlation with the storage temperature (Ding et al., 1998a). 1-MCP treatment could effectively maintain higher level of malic acid (Cao et al., 2011). In addition, citric acid and succinic acid were maintained relatively constant, while fumaric acid content in the fruit stored at 20°C was higher than that in the fruit stored at lower temperature (Ding et al., 1998a). The declining rate of organic acids was faster than that of sugar, which consequently elevated the ratio between sugars and acids, resulting in a flat flavor unsuitable for eating. Total carotenoids in loquat fruit increased steadily, and low temperature was not beneficial for the accumulation of carotenoids (Ding et al., 1998a). Chen et al. (2015) reported that lutein, β-cryptoxanthin, and β-carotene contents in “Zaozhong No.6” fruits stored at 25°C were higher than those at 4 and 8°C. Total phenolic content declined significantly during storage at 20°C. Ding et al. (1999) reported that the contents of chlorogenic acid, 5-feruloylquinic acid, neochlorogenic acid, and hydroxybenzoic acid significantly decreased during storage, whereas caffeic acid notably increased. Total flavonoids firstly increased and then decreased during cold storage (Cao et al., 2011).

Lignification is one of the important characteristics occurring naturally along with fruit ripening and senescence, which has been considered to be a major challenge confronted by loquat postharvest industry (Cai et al., 2006b), particularly in yellow-fleshed loquat. Low temperature storage promoted the incidence of flesh lignification, which was characterized by difficult peeling, browning, leathery, and juiceless pulp (Cai et al., 2006b; Li et al., 2017). Lignin is one of the ultimate metabolites produced by phenylpropanoid pathway, and its accumulation directly leads to lignification in plants (Zhao, 2016). Phenylpropanone is first deaminated, followed by a series of reactions, including hydroxylation, methylation, and reduction, and finally generates the lignin (Zhao, 2016). Genes encoding phenylalanine ammonia-lyase (PAL), cinnamate 4-hydroxylase (C4H), cinnamyl alcohol dehydrogenase (CAD), peroxidase (POD), and other key enzymes involved in lignin biosynthesis had been almost exclusively identified in plants. In addition, the activities of PAL, CAD, and POD were closely related to the lignification in loquat fruit. Cai et al. (2006b) found that lignification led to an increase in firmness of loquat fruit during storage at 20°C, which was resulted from increases in PAL, CAD, and POD activities. Shan et al. (2008) reported that the transcript abundance and the enzymatic activities of CAD and POD were strongly associated with lignification in loquat fruit. Moreover, the activity of CAD and the expression of *EjCAD1* were positively correlated with low temperature-induced lignification in loquat fruit. Additionally, other genes also displayed important roles in lignification of loquat. *EjCCoAOMT* was induced by low temperature, and this might be another reason for lignification stored at low temperature (Liu et al., 2015b). The activities of PAL, C4H, and 4CL were positively correlated with lignification of loquat fruit (Li et al., 2017). All results suggest that various enzymes involved in phenylpropanoid pathway contribute to lignin biosynthesis in loquat fruit during storage. Recently, several transcription factors have been proved to directly regulate lignin biosynthesis. EjMYB1 and EjMYB2 can competitively interact with the promoter of lignin biosynthetic genes to regulate loquat fruit lignification in chilling injury (Xu et al., 2014b). EjNAC1 could activate the expression of genes involved in lignin biosynthesis pathway (Xu et al., 2015). EjAP2–1 could interact with EjMYBs and function as an indirect transcriptional repressor on fruit lignification induced by chilling injury (Zeng et al., 2015). EjHSF3 could regulate loquat fruit lignification *via* interaction with lignin biosynthetic genes and the regulator EjAP2–1 (Zeng et al., 2016). EjMYB8 served as a regulator in chilling-induced lignification of loquat fruit by physically binding to *Ej4CL1* promoter, and transient overexpression of *EjMYB8* in tobacco and loquat leaves increased the lignification level (Wang et al., 2016). EjNAC3 could regulate chilling-induced lignification of loquat fruit *via* direct interaction with an atypical CAD-like gene (Ge et al., 2017). All of these transcription factors significantly modulated the expression of genes involved in phenylpropanoid pathway for lignification during loquat fruit development and low temperature storage.

## Postharvest Technologies of Loquat Fruit

**Figure 4:**
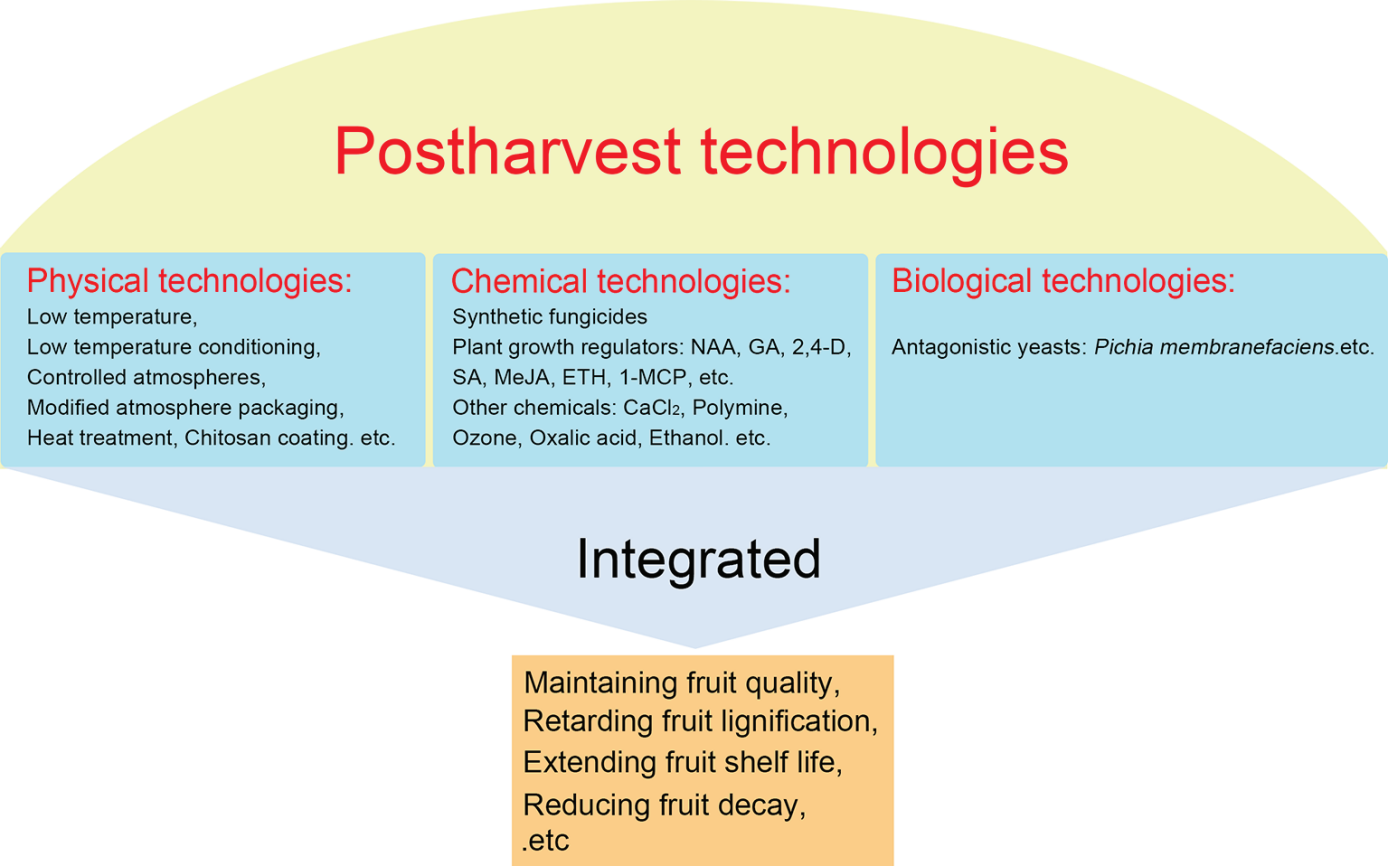
Postharvest technologies of loquat fruit.

Loquat fruit are perishable and have a short postharvest life at ambient temperature. Up to now, considerable strategies are currently available to reduce postharvest losses, maintain fruit quality, and prolong shelf life of harvested loquat fruit, which can be classified into three categories, namely physical, chemical, and biological technologies (Figure 4).

### Physical Technologies

Low temperature and controlled atmospheres are main physical measures to maintain the quality of fresh fruits and vegetables during storage (Tian et al., 2005; Wang et al., 2005; Meng et al., 2009; Zhang and Tian, 2010). In addition, other promising technologies, including hot treatment (Zong et al., 2010), radiation and microwave (Casals et al., 2010), hyperbaric pressure (Goyette et al., 2012), and ultraviolet radiation (Fonseca and Rushing, 2008), could also effectively maintain the quality of fruits. Like other fruits, low temperature storage is a technique widely used in the storage, transport, and market of loquat fruit. Low temperature could effectively decrease the respiration rate and ethylene production, extend shelf life, and reduce decay of harvested loquat fruit (Zheng et al., 1993; Ding et al., 1998a; Gao et al., 2007). We found that loquat fruit kept in modified atmosphere packaging (MAP), in which the gas compositions can be adjusted by fruit and vegetables respiration, resulting in high-CO_2_ and low-O_2_ atmospheres, at 1°C showed more effectiveness in reducing fruit decay, off-flavor, and weight loss than that at 6°C (Ding et al., 2006). The combination of 1-MCP and MAP displayed a better effect on maintaining the fruit quality by decreasing softening, deterioration, and browning rate (Oz and Ulukanli, 2011). However, extremely low temperature may cause chilling injury, resulting in increased firmness and lignification during storage, which was characterized by stuck peel, leathery, and juiceless pulp. 1-MCP treatment could effectively alle*via*te loquat fruit chill injury by modifying the fatty acid and cell wall polysaccharide composition and inhibiting LOX and phospholipase activities (Cao et al., 2009b,c). Additionally, low temperature conditioning (LTC) serves as an alternative technique for significantly improving tolerance to low temperature (Wang, 1993). LTC treatment (pre-stored at 10°C for 6 days then transferred to 0°C storage) could effectively reduce incidence of chilling injury and browning index, increase the content of glycine betaine, and maintain the content of sugar and titratable acids (Jin et al., 2015). Moreover, LTC treatment (pre-stored at 5°C for 6 days then transferred to 0°C storage) could also suppress the expression of some positive transcription factors and key genes involved in lignification of loquat fruit (Xu et al., 2014b; Wang et al., 2016).

Controlled atmospheres (CA) combined with low temperature storage are a more effective approach to maintain the quality and prolong the shelf life of loquat fruit. Our previous results indicated that loquat fruit kept in CA with 10% O_2_ plus 1% CO_2_ at 1°C could be stored for more than 50 days with normal flavor and low decay index (Ding et al., 2006). Moreover, short-term high-O_2_ treatment at the beginning of storage had little effect on fruit flavor, but stimulated ethanol accumulation in loquat fruit, and reduced activities of endo-polygalacturonase and exo-polygalacturonase (Ding et al., 2006). Treatment with high O_2_ (90%) could effectively inhibit the respiration rate and the PPO activity, which had a better flavor than control fruits after storage for 35 days (Zheng et al., 2000a). CA conditions are more effective for reducing the activities of PPO and oxidative stress compared to other treatments, such as LTC or MAP, which may be the reason why loquat fruit stored in CA conditions had lower decay index than that kept in other conditions.

Compared to low temperature and controlled atmosphere storage, other physical technologies applied in loquat fruit are scarce. Heat air treatment with 38°C for 5 h could dramatically reduce the internal browning of “Jiefangzhong” loquat fruit in chilling injury *via* maintenance of the integrity of cell membrane and higher unsaturated/saturated fatty acid ratio (Rui et al., 2010). Chitosan coating could significantly reduce weight loss and flesh browning, maintain antioxidant capacity, and minimize the losses of total polyphenol, carotenoid, and ascorbic acid in loquat fruits during cold storage (Petriccione et al., 2015).

### Chemical Technologies

Chemical technologies are still effective in controlling postharvest decay in fresh fruits and vegetables. In the early years, fungicides are widely applied in harvested loquat fruit to control postharvest diseases. Based on concerns about the safety of chemical fungicides, some plant growth regulators have been used to harvest loquat fruit. Treatment with salicylic acid (0.1 g/l) inhibited the rotten fruit, decreased the weight losses, and maintained higher titratable acid and ascorbic acid content of loquat fruit (Chen et al., 2008). Loquat fruit treated with methyl jasmonate (MeJA), which is a methyl ester widely distributed in plants, displayed significantly higher levels of sugars, organic acids, total phenolics, and carotenoids and maintained higher levels of antioxidants and antioxidant activity (Cao et al., 2009a). The mode of action of MeJA to alleviate chilling injury and enhance fruit resistance has been proved *via* enhancing the activities of antioxidant enzymes and PR-proteins expression (Cao et al., 2008a; Zhu and Tian, 2012; Jin et al., 2014). Exogenous 1-MCP treatment displayed longer shelf life and lower chilling injury by keeping lower respiration rate, PAL and LOX activities, and firmness (Ding et al., 2006; Cai et al., 2006a).

With the development of postharvest technologies, the role of many chemical agents applied in postharvest fruits and vegetables was further investigated in harvested loquat fruit. Calcium chloride treatment retained TSS and ascorbic acid content and decreased browning index, relative electrical conductivity, and weight loss (Babu et al., 2015). Ozone treatment at 150 mg/m^3^ had the best effect on loquat fruit and 200 mg/m^3^ treatment had obvious side effects with high decay (Zhang et al., 2011). Ethanol treatment could significantly reduce the disease incidence of anthracnose rot in harvested loquat by increasing the activities of PAL, POD, PPO, chitinase, and β-1,3-glucanase and inhibiting the growth of *Colletotrichum acutatum* (Wang et al., 2015b). However, chemical technologies have an obvious effect on extending the storage life of loquat fruit, and the regulation of chemicals has a concentration effect. The optimum concentration depended on loquat cultivars, fruit maturity, treatment time, and temperature of storage environment.

### Biological Technologies

Biological technologies, serving as healthy and environmentally friendly method for controlling disease, have been widely considered as potential methods instead of chemical fungicides in postharvest field. Currently, antagonistic yeasts have been proved to effectively control postharvest decay of various fruits, such as tomato (Zong et al., 2010), peach (Xu et al., 2008), sweet cherry (Xu and Tian, 2008), loquat (Cao et al., 2008b), table grape (Meng and Tian, 2009), apple (Li et al., 2016a), and banana (Zhimo et al., 2017). Antagonistic yeasts have a wide range of advantages, including broad-spectrum antimicrobial, simply nutrition requirement, easy culture, strong resistance to biotic and abiotic stresses, and flexible and easy to combine with other methods (Liu et al., 2011). Cao et al. (2008b) found that treatment with *Pichia membranifaciens* at 1 × 10^8^ CFU ml^−1^ significantly inhibited the incidence and progress of anthracnose rot in the harvested loquat fruit. However, application of antagonistic yeasts could not gain a satisfying result as fungicides. Many attempts have been made to increase the efficacy of antagonistic yeasts in controlling postharvest decay, especially combination with other treatments, such as calcium chloride (An et al., 2012), silicon (Qin and Tian, 2005), glycine betaine (Liu et al., 2011), salicylic acid (Qin et al., 2003), chitosan (Meng and Tian, 2009), and MeJA (Yao and Tian, 2005). Application of MeJA (Cao et al., 2009c) and CaCl_2_ (Cao et al., 2008b) could effectively improve the biocontrol activity of *P. membranifaciens* on anthracnose rot in harvested loquat fruit. These integrative techniques will be used to effectively control postharest diseases of loquat fruit.

## Author Contributions

JC, TC, ZZ, BL, GQ, and ST designed and wrote the manuscript.

### Conflict of Interest Statement

The authors declare that the research was conducted in the absence of any commercial or financial relationships that could be construed as a potential conflict of interest.
